# Immune cells phenotype and bioenergetic measures in CD4^+^ T cells differ between high and low feed efficient dairy cows

**DOI:** 10.1038/s41598-024-66345-x

**Published:** 2024-07-10

**Authors:** Usman Arshad, Katherine M. Kennedy, Malena Cid de la Paz, Sophia J. Kendall, Lautaro R. Cangiano, Heather M. White

**Affiliations:** Department of Animal and Dairy Sciences, University of Wisconsin-Madison, 1675 Observatory Drive Rm 952D, Madison, WI 53706 USA

**Keywords:** Immunology, Physiology

## Abstract

Identifying sources of variance that contribute to residual feed intake (RFI) can aid in improving feed efficiency. The objectives of this study were to investigate immune cells phenotype and bioenergetic measures in CD4^+^ T cells in low feed efficient (LE) and high feed efficient (HE) dairy cows. Sixty-four Holstein cows were enrolled at 93 ± 22 days in milk (DIM) and monitored for 7 weeks to measure RFI. Cows with the highest RFI (LE; n = 14) or lowest RFI (HE; n = 14) were selected to determine immune cells phenotype using flow cytometry. Blood was sampled in the same LE and HE cows at 234 ± 22 DIM to isolate peripheral blood mononuclear cells, followed by magnetic separation of CD4^+^ T lymphocytes using bovine specific monoclonal antibodies. The metabolic function of isolated CD4^+^ T lymphocytes was evaluated under resting and activated states. An increased expression of CD62L^+^ cells within CD8^+^ T lymphocytes and CD21^+^ B lymphocytes was observed in HE cows compared to LE cows. CD4^+^ T lymphocytes of HE cows exhibited an increased mitochondrial and glycolytic activity in resting and activated states compared to LE cows. These data suggest that immune cells in HE cows exhibit an increased metabolic function, which might influence nutrient partitioning and utilization and serve as a source of variation in feed efficiency that warrants future investigation.

## Introduction

Residual feed intake (RFI) is one method of quantifying feed efficiency and is calculated based on the difference between observed and predicted dry matter intake (DMI) after adjusting for multiple energy sinks^1,2^. High feed efficient (HE) cows have negative RFI values as they consume less dry matter (DM) to produce the same amount of milk compared to others in their cohort after adjusting for known energy sinks^2,3^. Identifying sources of variance can both reduce the “unknown” RFI fraction^4^ and allow for improvements in animal feed efficiency by targeting these newly identified energy sinks. One potential energy sink contributing to RFI that is not currently accounted for is the immune system. Immune response is nuanced, and immune activation should be considered on a continuum of activity, rather than as activated or not^5^. Having a robust and active immune system is likely key to maintaining health and efficiency and has been described as “immune readiness” in humans^6^ and rodents^7^. An experiment was conducted to determine glucose requirements of an activated immune system in lactating Holstein cows and demonstrated that an activated immune system in a lactating cow consumes > 1 kg of glucose within 12 h of an acute infection^8^.

Increased glucose utilization by the activated immune system is reflective of the increased glucose uptake necessary during immune activation^9,10^. In the case of T cells, resting CD4^+^ T cells rely on oxidative phosphorylation, but activation through T-cell receptor and costimulatory receptors such as CD4 and CD28 enhances glucose uptake, triggering intracellular signaling for increased mitochondrial respiration and glycolysis^9,11^. This metabolic switch in activated CD4^+^ T cells enables the cells to utilize mitochondrial respiration, which upregulates the synthesis of biomolecules required during clonal expansion^12,13^. Additionally, increased glycolytic activity within activated CD4^+^ T cells isolated from dairy cows was also observed^14^, which indicates that glucose is essential for cellular growth and proliferation. Nevertheless, the information with reference to divergent RFI and metabolic function of CD4^+^ T cells in resting and activated states is scarce in lactating dairy cows.

Considering the substantial glucose utilization by immune cells during an immune response, it is possible that cows with different feed efficient status could partition nutrients differently, ultimately influencing feed efficiency and RFI. Preliminary evidence indicated that HE mid-lactating cows showed an increased expression of genes associated with both innate and adaptive immune responses in liver and muscle tissues compared to least feed efficient (LE) cows based on RNA sequencing analysis^15^. This work proposed that HE cows might exhibit a phenomenon of immune readiness even in the healthy state; however, the activity of immune cells in high and low RFI cows and how it potentially contributes to energy sinks and feed efficiency were not directly evaluated in that study, warranting further investigation^15^. Ferronato et al.^16^ investigated the relationship between divergent RFI and immunometabolism in healthy Italian Simmental calves and demonstrated that HE calves had reduced blood markers related to oxidative stress and systemic inflammation compared to LE calves, supporting that the immune system might be a potential component to explain variability in feed efficiency in dairy cows.

We hypothesized that immune cells in HE cows would exhibit a phenotype resembling increased metabolic function compared to LE cows. Therefore, the objectives of this study were to (1) investigate the immune cells phenotype, as well as the phagocytic and oxidative burst activities, in neutrophils in LE and HE dairy cows and (2) assess the in vitro metabolic function of CD4^+^ T lymphocytes in resting and activated states according to divergent RFI status in dairy cows.

## Results

### Production responses according to feed efficiency

The ingredients and chemical composition of the diet is presented in Table 1. Production and energy characteristics for LE and HE cows are presented in Table 2. Cows identified as LE had a positive RFI, whereas cows identified as HE had a negative RFI (LE = 1.93 vs. HE = – 1.76 ± 0.19 kg/d; *P* < 0.0001) primarily because HE cows had reduced DMI compared to LE cows (*P* = 0.001). The net energy for lactation (NE_L_) intake was greater in LE cows compared to HE cows (*P* = 0.001); nevertheless, the milk and energy-corrected milk yields and milk components did not differ between LE and HE cows (*P* ≥ 0.78). Body weight and body condition score did not differ between LE and HE cows (*P* ≥ 0.52).

**Table 1:** Ingredients and nutrient composition of diet.

Item	Herd diet
Ingredient, % of diet DM	
Corn silage	28.6 ± 1.21
Alfalfa silage	23.8 ± 1.32
Concentrate^1^	28.0 ± 0.70
High moisture corn	12.7 ± 0.83
Cottonseed	4.7 ± 0.41
Distiller’s grains	2.2 ± 0.15
Nutrient composition, % of diet DM	
DM	52.3 ± 1.01
CP	17.3 ± 0.06
ADF	21.6 ± 0.40
NDF	28.3 ± 0.45
Starch	24.3 ± 0.75
Ether extract	4.7 ± 0.06
Ash	7.8 ± 0.08
Net energy of lactation, Mcal/kg	1.51

^1^Concentrate was formulated to contain (% as fed): 26.4% ground shell corn; 18.1% soy hulls; 15.6% canola meal; 17.3% soybean meal; 11.6% expeller meal (SoyPlus^®^, Landus Cooperative); 4.6% calcium carbonate; 2.5% sodium bicarbonate; 1.3% mineral mix [% as fed; 82.12% NaCl; 0.35% Ca; 0.09% S; 0.01% Co; 0.38% monensin; 0.48% Cu; 0.04% I; 0.15% Fe; 1.43% Mn; 0.01% Se; 2.05% Zn; 0.004% biotin; 0.07% diflubenzuron; 1.954 kIU/kg vitamin E; 391 kIU/kg vitamin A; 78 kIU/kg vitamin D_3_]; 0.8% magnesium oxide (56%); 0.6% urea (46%); 0.3% potassium carbonate; 0.3% tallow (MaxFat, Sanimax Corp.); 0.3% yeast culture (Celmanax™ Dry, Church & Dwight Co., Inc.); 0.2% Smartamine M (Adisseo USA Inc.); 0.2% probiotic (Fortress LG, Vita Plus Corp.); and 0.2% mineral supplement (Dynamate, Pestell Nutrition Inc.).

**Table 2:** Production, reproduction, and energy characteristics according to feed efficiency status in mid-lactating dairy cows.

Item	Feed efficiency group^1^		SEM	*P*-value
	LE	HE		RFI
Productive performance^2^				
Residual feed intake, kg/d	1.93	− 1.76	0.19	< 0.0001
Dry matter intake, kg/d	32.9	29.3	0.70	0.001
NE_L_ intake^3^, Mcal/d	49.7	44.3	1.06	0.001
Body weight, kg	729	746	19.2	0.54
Body condition score, 1 to 5	3.18	3.22	0.04	0.52
Yield, kg/d				
Milk	50.5	50.6	2.62	0.98
Fat	2.02	2.00	0.07	0.88
Protein	1.57	1.58	0.06	0.94
Lactose	2.40	2.45	0.13	0.78
Energy-corrected milk	53.5	53.5	2.15	0.99
Reproductive performance^4^				
Pregnant^5^, % (n/n)	83.3 (10/12)	91.7 (11/12)	9.4	0.55
Services per conception^6^, n	1.75	1.67	0.28	0.84
Days open^7^, n	111	107	11	0.79

^1^Residual feed intake (RFI) was calculated at week 7 relative to enrollment based on the difference between observed and predicted dry matter intake, and cows were categorized into least feed efficient (LE) or high feed efficient (HE) group.

^2^Productive performance of 28 cows (LE; n = 14, HE; n = 14) was monitored between mean (± standard deviation) 93 ± 22 and 149 ± 25 days in milk (DIM).

^3^NE_L_ intake = intake of net energy of lactation.

^4^Reproductive performance of 24 cows (LE; n = 12, HE; n = 12) was monitored at 234 ± 22 DIM.

^5^Indicates pregnancy per artificial insemination (AI) for all AI performed during the lactation cycle. Data on pregnancy was presented based on ultrasonography diagnosis at day 70 after insemination.

^6^Total number of inseminations performed to achieve a pregnancy.

^7^Total number of days in which cows did not carry a pregnancy.

### Phenotype of B and T lymphocytes by feed efficiency category

The proportion of T and B lymphocytes and expression of their receptors are presented in Table 3. The expression of CD8^+^ T lymphocytes tended to be greater in HE compared to LE cows (*P* = 0.07). The median fluorescence intensity (MFI) of CD62L^+^ cells within CD8^+^ T lymphocytes was greater in HE compared to LE cows (*P* < 0.0001). On the contrary, the proportion of CD21^+^ T lymphocytes tended to be reduced in HE cows compared to LE cows (*P* = 0.08). In HE cows, there was a trend toward a greater proportion of CD62L^+^ lymphocytes compared to LE cows (*P* = 0.08). Additionally, the expression of CD62L^+^ cells within CD21^+^ B lymphocytes was significantly greater in HE cows than in LE cows (*P* = 0.02). Similarly, there was a tendency for a greater proportion of CD62L^+^ cells within γ-delta T lymphocytes in HE cows compared to LE cows (*P* = 0.06).

**Table 3:** Evaluation of T and B lymphocytes panels according to feed efficiency status in mid-lactating dairy cows.

Item^2^	Feed efficiency group^1^		SEM	*P*-value
	LE	HE		RFI
Live cells^3^, % of lymphocytes	40.1	37.6	3.2	0.58
CD4^+4^, %	20.4	22.3	2.0	0.52
CD4^+5^, MFI	728	690	70.3	0.70
CD62L^+6^, %	8.52	7.83	1.24	0.70
CD62L^+7^, MFI	443	450	5.80	0.42
CD8^+8^, %	9.95	9.78	0.92	0.89
CD8^+9^, MFI	2025	2464	166	0.07
CD62L^+10^, %	7.73	9.09	1.15	0.41
CD62L^+11^, MFI	569	608	11.2	< 0.0001
CD21^+12^, %	37.3	32.0	2.1	0.08
CD21^+13^, MFI	10,122	9835	670	0.76
CD62L^+14^, %	3.22	4.48	0.49	0.08
CD62L^+15^, MFI	637	653	4.5	0.02
GDT^+16^, %	11.6	13.5	1.8	0.48
GDT^+17^, MFI	416	409	22.7	0.83
CD62L^+18^, %	26.9	34.5	3.4	0.06
CD62L^+19^, MFI	395	424	28.5	0.47

^1^Residual feed intake was calculated at week 7 relative to enrollment based on the difference between observed and predicted dry matter intake, and cows were categorized into least feed efficient (LE) or high feed efficient (HE) group.

^2^Mononuclear cells, i.e. lymphocytes were identified based on size and cell complexity.

^3^Percentage of live lymphocytes within the total live cells population.

^4^Percentage of CD4^+^ T cells within the live lymphocyte population.

^5^Median fluorescence intensity of CD4^+^ T cells.

^6^Percentage of CD62L^+^ cells within CD4^+^ T cells.

^7^Median fluorescence intensity of CD62L^+^ cells within CD4^+^ T cells.

^8^Percentage of CD8^+^ T cells within the live lymphocyte population.

^9^Median fluorescence intensity of CD8^+^ T cells.

^10^Percentage of CD62L^+^ cells within CD8^+^ T cells.

^11^Median fluorescence intensity of CD62L^+^ cells within CD8^+^ T cells.

^12^Percentage of CD21^+^ B cells within the live lymphocyte population.

^13^Median fluorescence intensity of CD21^+^ B cells.

^14^Percentage of CD62L^+^ cells within CD21^+^ B cells.

^15^Median fluorescence intensity of CD62L^+^ cells within CD21^+^ B cells.

^16^Percentage of γ-delta^+^ T cells within the live lymphocyte population.

^17^Median fluorescence intensity of γ-delta^+^ T cells.

^18^Percentage of CD62L^+^ cells within γ-delta^+^ T cells.

^19^Median fluorescence intensity of CD62L^+^ cells within γ-delta^+^ T cells.

### Phenotype of monocytes and neutrophils function according to feed efficiency

The proportion of monocytes and expression of their receptors and phagocytosis and oxidative burst capacities in neutrophils are presented in Table 4. The expression of CD14^+^ cells within CD11b^+^ monocytes tended to be greater in HE cows compared to LE cows (*P* = 0.07). The percentage of phagocytic neutrophils or MFI of phagocytosis and oxidative burst in neutrophils did not differ between LE and HE dairy cows (*P* ≥ 0.28).

**Table 4:** Evaluation of monocytes panels and neutrophils functions according to feed efficiency status in mid-lactating dairy cows.

Item	Feed efficiency group^1^		SEM	*P*-value
	LE	HE		RFI
Mononuclear cells^2^				
CD11b^+3^, %	16.2	18.9	1.9	0.33
CD11b^+4^, MFI	11,775	12,010	493	0.74
CD14^+5^, %	82.4	85.2	2.2	0.38
CD14^+6^, MFI	2805	3141	128	0.07
CD62L^+7^, %	10.7	10.0	1.3	0.70
CD62L^+8^, MFI	281	279	1.3	0.50
Granulocytes^9^				
Phagocytosis^10^, %	33.7	34.0	2.5	0.92
Phagocytosis^11^, MFI	37,823	33,446	2828	0.28
Oxidative burst^12^, MFI	16,142	15,499	1659	0.79

^1^Residual feed intake was calculated at week 7 relative to enrollment based on the difference between observed and predicted dry matter intake, and cows were categorized into least feed efficient (LE) or high feed efficient (HE) group.

^2^Mononuclear cells i.e. monocytes were identified based on size and cell complexity.

^3^Percentage of CD11b^+^ cells (monocytes) within the total live population of monocytes.

^4^Median fluorescence intensity of CD11b^+^ monocytes.

^5^Percentage of CD14^+^ cells within CD11b^+^ monocytes.

^6^Median fluorescence intensity of CD14^+^ cells within CD11b^+^ monocytes.

^7^Percentage of CD62L^+^ cells within CD11b^+^ monocytes.

^8^Median fluorescence intensity of CD62L + cells within CD11b^+^ monocytes.

^9^Granulocytes i.e. neutrophils were identified based on size and cell complexity.

^10^Percentage of phagocytic neutrophils.

^11^Median fluorescence intensity of neutrophils for phagocytosis.

^12^Median fluorescence intensity of neutrophils for oxidative burst.

### Mitochondrial and glycolytic respiration according to feed efficiency category

**Figure 1:**
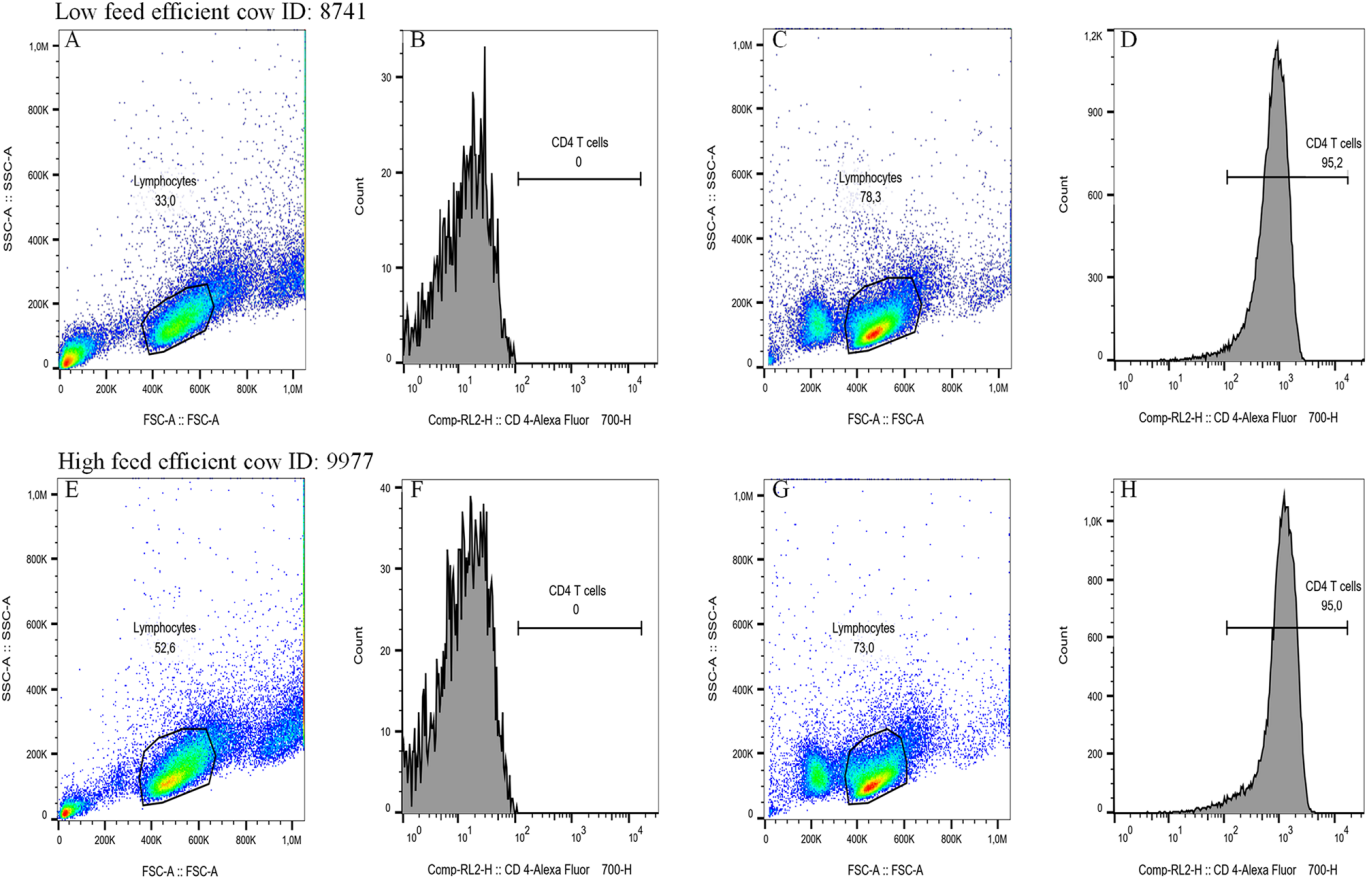
Representation of purification of CD4^+^ T lymphocytes after their isolation from representative least feed efficient (LE) and high feed efficient (HE) mid-lactating dairy cows. The CD4^+^ T lymphocytes were isolated by magnetic cell separation from peripheral blood mononuclear cells (PBMC), which were assessed using flow cytometer. Lymphocytes were identified using forward scatter (FSC) and side scatter (SSC) parameters. Within the live lymphocytes gated population, CD4^+^ T lymphocytes were gated using anti-CD4^+^ antibody. Panels (**A**) and (**B**) and panels (**E**) and (**F**) indicate that PBMC were not incubated with anti-CD4^+^ antibody and served as a control group. Panels (**C**) and (**D**) and panels (**G**) and (**H**) represent that PBMC were incubated with an anti-CD4^+^ antibody conjugated with Alexa fluor 700 and served as a positive group. The CD4^+^ T lymphocytes were identified by gating the lymphocyte population (Panels (**A**) and (**B**) and Panels (**E**) and (**F**)), followed by CD4^+^ T lymphocytes expression in channel RL2 (Panels (**C**) and (**D**) and Panels (**G**) and (**H**)). The control group contained 0% of CD4^+^ T lymphocytes (Panel **B**), and the positive group represented 95.2% of CD4^+^ T lymphocytes (Panel **D**) in LE cows, whereas the positive group contained 0% of CD4^+^ T lymphocytes (Panel **F**), and the positive group represented 95.0% of CD4^+^ T lymphocytes (Panel **H**) in HE cows.

**Figure 2:**
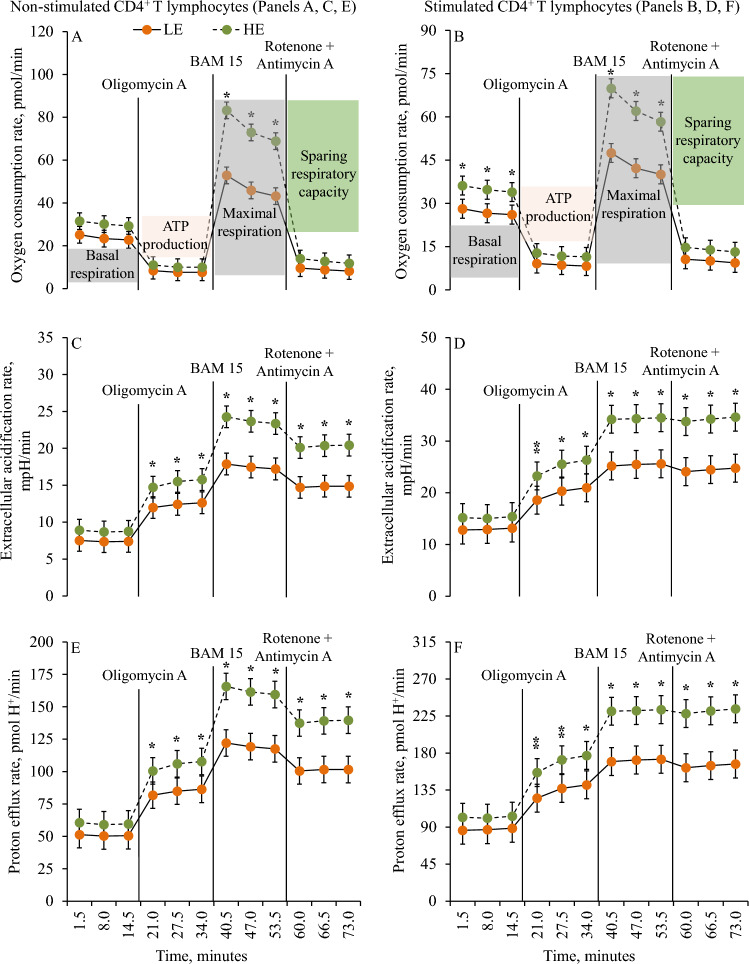
Mitochondrial and glycolytic functional kinetics are presented in non-stimulated and stimulated CD4^+^ T lymphocytes according to divergent residual feed intake (RFI) in mid-lactating dairy cows. Cows were ranked according to RFI after 7 weeks relative to enrollment and categorized as least feed efficient (LE) and high feed efficient (HE). Oxygen consumption rate (OCR), extracellular acidification rate (ECAR), and proton efflux rate (PER) are presented in non-stimulated (Panels **A**, **C**,** E**) and stimulated (Panels **B**, **D**, **F**) CD4^+^ T lymphocytes in LE and HE dairy cows. Mitochondrial and glycolytic functional kinetics were recorded in real-time measuring OCR and ECAR, respectively, under basal conditions and in response to complex V inhibitor (oligomycin), a protonophore uncoupler (BAM 15), and complex I and complex III inhibitors (rotenone and antimycin A) to evaluate bioenergetic measures such as basal respiration, maximal respiration, ATP production, and sparing respiratory capacity. Panel (**A**) association of feed efficiency (*P* = 0.001), time (*P* < 0.0001), and interaction between feed efficiency ⨉ time (*P* < 0.0001). Panel (**B**) association of feed efficiency (*P* = 0.001), time (*P* < 0.0001), and interaction between feed efficiency ⨉ time (*P* < 0.0001). Panel (**C**) association of feed efficiency (*P* = 0.002), time (*P* < 0.0001), and interaction between feed efficiency and time (*P* < 0.0001). Panel (**D**) association of feed efficiency (*P* = 0.02), time (*P* < 0.0001), and interaction between feed efficiency ⨉ time (*P* < 0.0001). Panel (**E**) association of feed efficiency (*P* = 0.002), time (*P* < 0.0001), and interaction between feed efficiency ⨉ time (*P* < 0.0001). Panel (**F**) association of feed efficiency (*P* = 0.02), time (*P* < 0.0001), and interaction between feed efficiency ⨉ time (*P* < 0.0001). Symbols indicate level of significance (* = *P* ≤ 0.05; ^⁑^0.05 < *P* ≤ 0.10). Error bars depict standard errors of the means.

**Figure 3:**
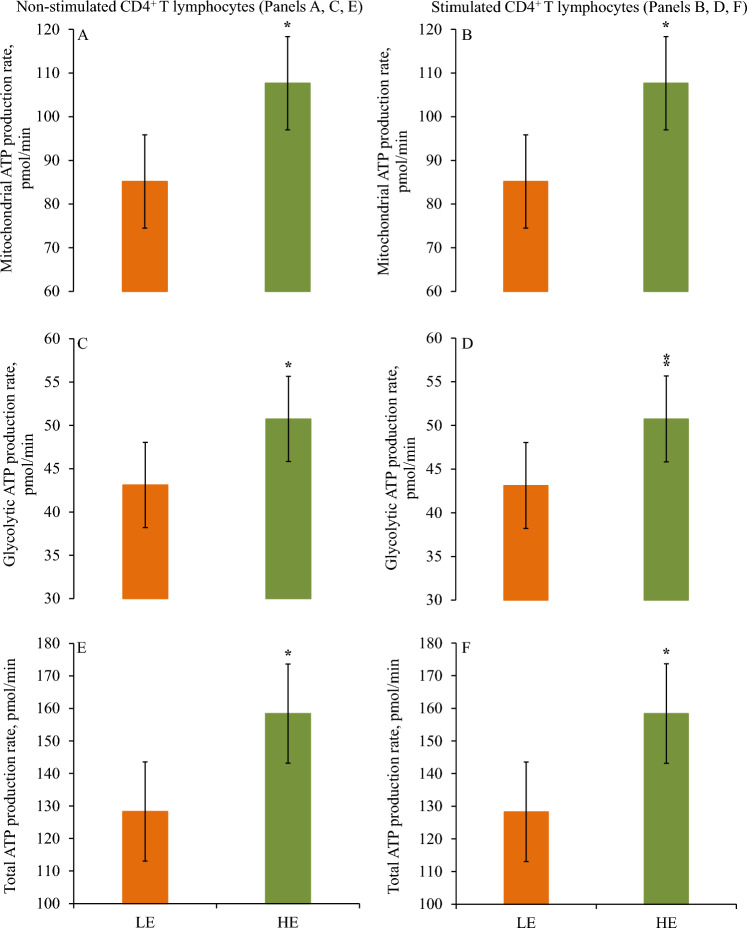
Mitochondrial and glycolytic ATP production rates are presented in non-stimulated and stimulated CD4^+^ T lymphocytes according to divergent residual feed intake (RFI) in mid-lactating dairy cows. Cows were ranked according to RFI after 7 weeks relative to enrollment and categorized as least feed efficient (LE) and high feed efficient (HE). Mitochondrial ATP production rate, glycolytic ATP production rate, and total ATP production rate are presented in non-stimulated (Panels **A**, **C**,** E**) and stimulated (Panels **B**,** D**, **F**) CD4^+^ T lymphocytes in LE and HE dairy cows. Panel (**A**) association of feed efficiency (*P* = 0.01). Panel (**B**) association of feed efficiency (*P* = 0.02). Panel (**C**) association of feed efficiency (*P* = 0.03). Panel (**D**) association of feed efficiency (*P* = 0.09). Panel (**E**) association of feed efficiency (*P* = 0.01). Panel (**F**) association of feed efficiency (*P* = 0.02). Symbols indicate level of significance (* = *P* ≤ 0.03; ^⁑^0.05 < *P* ≤ 0.10). Error bars depict standard errors of the means.

The purity of CD4^+^ T lymphocytes (mean ± standard deviation) after isolation from peripheral blood mononuclear cells (PBMC) was 91.9 ± 2.8% and 92.8 ± 2.8% in LE and HE cows, respectively, and dot plots from representative LE and HE cows are presented in Fig. 1A to H. The oxygen consumption rate (OCR) in non-stimulated CD4^+^ T lymphocytes was affected by the interaction (*P* < 0.0001) between feed efficiency and time (Fig. 2A). The interaction was mainly attributed to the greater increase in mitochondrial respiration in HE cows compared to LE after CD4^+^ T lymphocytes were exposed to an uncoupler (BAM 15). Similarly, OCR in stimulated CD4^+^ T lymphocytes was also affected by the interaction between feed efficiency and time (*P* < 0.0001; Fig. 2B). In the case of metabolically active CD4^+^ T lymphocytes, the basal respiration and mitochondrial respiration were both greater in HE compared to LE cows (Fig. 2B). The extracellular acidification rate (ECAR) in non-stimulated and stimulated CD4^+^ T lymphocytes increased progressively over time in HE compared to LE cows (*P* < 0.0001; Fig. 2C and D, respectively). Similarly, the proton efflux rate (PER) in non-stimulated and stimulated CD4^+^ T lymphocytes was also increased progressively over time in HE cows compared to LE cows (*P* < 0.0001; Fig. 2E and F). The basal mitochondrial ATP production rate in non-stimulated or stimulated CD4^+^ T lymphocytes was greater in HE cows compared to LE cows (*P* ≤ 0.02; Fig. 3A and B). Similarly, the basal glycolytic ATP production rate in non-stimulated CD4^+^ T lymphocytes was greater in HE cows compared to LE cows (*P* = 0.03; Fig. 3C). The basal glycolytic ATP production rate in stimulated CD4^+^ T lymphocytes tended to be greater in HE cows compared to LE cows (*P* = 0.09; Fig. 3D). Irrespective of the metabolic state (resting or activated) of CD4^+^ T lymphocytes, the total basal ATP production rate was greater in HE cows compared to LE cows (*P* ≤ 0.02; Fig. 3E and F).

**Figure 4:**
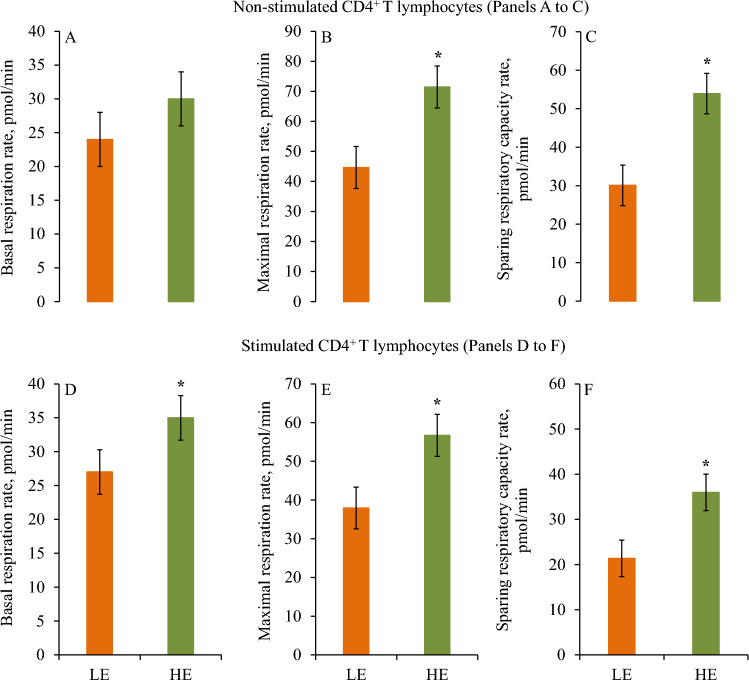
Bioenergetic measures in non-stimulated and stimulated CD4^+^ T lymphocytes are presented according to divergent residual feed intake (RFI) in mid-lactating dairy cows. Cows were ranked according to RFI after 7 weeks relative to enrollment and categorized as least feed efficient (LE) and high feed efficient (HE). Basal respiration rate, maximal respiration rate, and sparing respiratory capacity rate are presented in non-stimulated (Panels (**A**) to (**C**)) and stimulated (Panels (**D**) to (**F**)) CD4^+^ T lymphocytes in LE and HE dairy cows. Panel (**A**) association of feed efficiency (*P* = 0.14). Panel (**B**) association of feed efficiency (*P* = 0.001). Panel (**C**) association of feed efficiency (*P* = 0.0006). Panel (**D**) association of feed efficiency (*P* = 0.03). Panel (**E**) association of feed efficiency (*P* = 0.02). Panel (**F**) association of feed efficiency (*P* = 0.04). Symbols indicate level of significance (* = *P* ≤ 0.03). Error bars depict standard errors of the means.

The basal mitochondrial respiration in non-stimulated CD4^+^ T lymphocytes did not differ between LE and HE cows (*P* = 0.14; Fig. 4A). Nevertheless, the maximal mitochondrial respiration in non-stimulated CD4^+^ T lymphocytes was greater in HE cows compared to LE cows (LE = 44.6 vs. HE = 71.4 ± 6.99 pmol/min; *P* = 0.001; Fig. 4B). The sparing respiratory capacity rate (SRCR) in non-stimulated CD4^+^ T lymphocytes was increased in HE cows compared to LE cows (*P* = 0.0006; Fig. 4C). The basal mitochondrial respiration in stimulated CD4^+^ T lymphocytes increased in HE cows compared to LE cows (LE = 16.7 vs HE = 20.7 ± 2.11 pmol/min; *P* = 0.03; Fig. 4D). Moreover, the maximal mitochondrial respiration in stimulated CD4^+^ T lymphocytes also increased in HE cows compared to LE cows (LE = 37.9 vs HE = 56.7 ± 5.42 pmol/min; *P* = 0.02; Fig. 4E). The SRCR in stimulated CD4^+^ T lymphocytes increased in HE cows compared to LE cows (*P* = 0.04; Fig. 4F).

### Relationships between residual feed intake and reproductive performance

All 24 cows in the second component of the study received at least one artificial insemination (AI) and a pregnancy diagnosis. Cows were subjected to timed AI for first AI, and no associations (*P* ≥ 0.55) were observed between feed efficiency and pregnancy per AI to all AI, services per conception, and days open in the postpartum period (Table 2).

### Relationships between residual feed intake and bioenergetic measures

**Figure 5:**
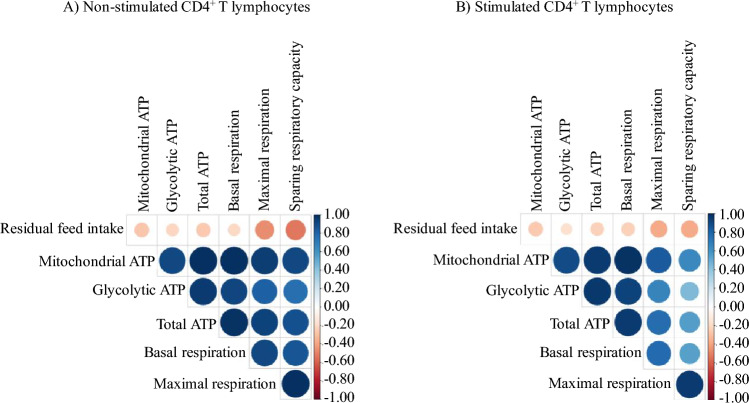
Pearson correlations between residual feed intake and bioenergetic measures are presented in non-stimulated (**A**) and stimulated (**B**) CD4^+^ T lymphocytes. The bioenergetic measures included mitochondrial ATP production rate, glycolytic ATP production rate, total ATP production rate, basal respiration rate, maximal respiration rate, and sparing respiratory capacity rate. Data is graphically presented in a dot plot heatmap. Strong correlations indicated by large circles and weaker correlations indicated by smaller circles. The scale colors denote whether the correlation is positive (closer to 1, blue circles) or negative (closer to − 1, red circles).

Increasing RFI was negatively associated with maximal mitochondrial respiration and SRCR in non-stimulated CD4^+^ T lymphocytes (-0.53 < r < -0.46; *P* ≤ 0.02; Fig. 5A). Increasing RFI was also correlated negatively with maximal mitochondrial respiration, and SRCR in stimulated CD4^+^ T lymphocytes (r = − 0.37; *P* ≤ 0.07; Fig. 5B).

## Discussion

Enhancing the immune response stands as a valuable trait to be selected for in dairy cattle, primarily due to its association with reduced disease occurrence, ultimately fostering improved health and enhanced production performance within the herd. Moreover, feed efficiency is also a desirable trait as it is strongly associated with increased farm profitability and reduced environmental impacts of dairy farming. The relationships between feed efficiency and immune function are not yet explored in dairy cows. In this study, we hypothesized that immune cells in HE cows would exhibit a phenotype resembling immune readiness. In humans and rodents, the concept of immune readiness is associated with a capacity for increased responsiveness, characterized by increased expression of pro-inflammatory cytokines and enhanced responsiveness to immune stimuli^6,7^. This state of immune readiness is believed to confer a protective advantage against pathogens and other challenges. The present study showed increased expression of receptors involved in T and B cells activation in healthy mid-lactating HE cows compared to LE cows. Furthermore, the metabolic activity observed in resting or stimulated CD4^+^ T lymphocytes in HE cows surpassed that of LE cows. Consequently, it is possible to speculate that selecting cows for improved feed efficiency based on RFI might carry a phenotype related to immune readiness, which facilitates mounting a faster or more efficient immune response.

In the current study, all cows were healthy throughout the study and during the sampling periods. The HE cows had an increased expression of coreceptor CD8 in CD8^+^ T cells, concurrent with an increased expression of CD62L^+^ cells within CD8^+^ T cells and CD21^+^ B cells, when compared to LE cows. The expression of CD62L^+^, also known as L-selectin, serves as a pivotal cell adhesion molecule present on the surface of specific immune cells, facilitating their migration towards sites of infection^17^. Moreover, its upregulation is indicative of cellular activation. In the context of HE cows, increased expression of CD62L^+^ across multiple lymphocyte subsets suggests a heightened readiness and responsiveness of these immune cells. This may imply that HE cows possess an enhanced capacity for rapid immune cell mobilization and subsequent immune response upon encountering infectious challenges. Such findings underscore the potential immunological advantages associated with feed efficiency in cattle, highlighting the intricate interplay between metabolic efficiency and immune function.

In a previous study conducted in our laboratory, healthy mid-lactating cows were categorized based on RFI into HE (low RFI = − 1.73 kg/d) or LE (high RFI =  + 1.72 kg/d), and RNA seq results indicated that the molecular control of gene expression in liver and muscle tissue were associated with immune function, and differed according to RFI ranking^15^. It was observed that several highly expressed genes involved in the activation of the innate and adaptive immune response were upregulated in HE cows (*LYZ*, *AOAH*, *CLEC7A*, *GIMAP8*, *GIMAP6*, *MARCO*, and *CD180*) compared to LE cows^15^. In the present study, the increased expression of CD14^+^ cells were observed within CD11b^+^ monocytes in HE cows compared to LE cows. The CD14^+^ and CD11b^+^ monocytes are cell surface markers found on different types of immune cells, particularly myeloid cells such as monocytes, macrophages, and some dendritic cells^17,18^. When CD14^+^ expression increases within CD11b^+^ monocytes, it often indicates activation or differentiation of these myeloid cells^17,18^. The increased expression of CD14^+^ within CD11b^+^ monocytes signify their readiness to respond to bacterial or pathogen-associated molecular patterns that are recognized by CD14^+^ cells. When these cells encounter pathogens or microbial components like lipopolysaccharide, the upregulation of CD14^+^ cells can lead to enhanced phagocytosis, cytokine production, and activation of these myeloid cells as part of the innate immune response^19^. Even though all mid-lactating cows were healthy in this study, an increased expression of T cells, B cells, or monocytes suggest a state of activation and readiness for migration to infected tissues, which plays a crucial role in mounting effective immune responses against pathogens or antigens, and these findings are aligned to gene expression data of Caputo et al.^15^. Studies conducted on low and high RFI pigs showed no discernible difference in immune response when exposed to a viral challenge^20^. Conversely, HE broiler chickens exhibited increased inflammatory responses alongside decreased adaptive immune responses when exposed to similar viral challenges^21^. Since the cows in this study were not subjected to an immune challenge, the differences observed between LE and HE cows might indicate a state of immune readiness in the absence of full activation. To our knowledge, immune cells phenotypes in other species have not been characterized in divergent RFI animals in the absence of an immune challenge, nor has it been examined in dairy cows in the presence of an in vivo immune challenge, therefore, it is difficult to compare across species without additional research.

The present study revealed that CD4^+^ T cells in HE cows during resting and activated states exhibited enhanced metabolic activity, specifically demonstrating increased mitochondrial respiration and cellular glycolysis when subjected to mitochondrial stress. An interesting aspect was that HE cows showed increased basal respiration (before inducing mitochondrial stress) but only in activated CD4^+^ T lymphocytes compared to LE cows. This finding demonstrates that HE cows showed an enhanced metabolic responsiveness to carry out essential cellular functions. Furthermore, increased mitochondrial and glycolytic ATP production rates, and enhanced SRCR, were observed in HE cows after inducing mitochondrial stress in resting or activated CD4^+^ T lymphocytes. Sparing respiratory capacity rate represents the mitochondrial capacity to meet additional energy demands, beyond the basal level, in response to acute cellular stress, and serves as a gauge of mitochondrial function^22^. Additionally, SRCR is closely linked to mitochondrial plasticity, allowing for bioenergetic adaptability in response to pathophysiological stressors, and plays a pivotal role in metabolic pathways governing cell proliferation, differentiation, and death, impacting both normal and cancerous cells^23^. Throughout the immune response, T cells exhibit dynamic changes in SRCR, with different T-cell subpopulations displaying distinct metabolic profiles. Evidence suggests that effector CD4^+^ T cells primarily rely on glycolysis for proliferation, differentiation, and survival, whereas regulatory T-cells predominantly utilize mitochondrial fatty acid oxidation, resulting in greater SRCR^24^. The increased SRCR in HE cows observed in this study may confer advantageous effects on immune function. Elevated SRCR could equip immune cells with greater metabolic flexibility and resilience, allowing for more efficient energy production and utilization in response to shifts in their environment^25^, including immune challenges, nutrient availability, and oxidative stress. Resiliency to such macro- or micro-metabolic changes may contribute to feed efficiency, and future work should explore the potential contribution of immune readiness to whole animal resiliency.

Considering the potential of HE cows to optimize their metabolic function, which may support synthesis of cytokines or chemokines concurrent with their rapid cell proliferation, it is reasonable to propose that HE cows may mount faster immune responses, thereby aiding in the maintenance of their health comparable to that of LE cows^26^. Indeed, it has been shown that dairy cows identified as high immune responders exhibited a reduced susceptibility to infectious diseases such as mastitis, metritis, and retained placenta^27^. Thus, it is possible that it might be more advantageous to increase generalized disease resistance through improved immune readiness, rather than focusing on the targeting of individual diseases or pathogens. Selecting dairy cows with higher breeding values for antibody and cell-mediated responses results in enhanced immune responsiveness, which may confer advantages in terms of disease resistance, pathogen clearance, and overall health. These high immune responder cows have been shown to exhibit faster and more efficient immune cell activation, enhanced antibody production, and improved immune surveillance, which could contribute to a more effective defense against infectious agents^28^. Additionally, a study focusing on cows during the peripartum period suggests potential benefits for milk yield in individuals displaying elevated immune responsiveness^29^. One aspect related to the activated immune response involves rapid proliferation of immune cells which involves increased utilization of glucose^8,30^, which may compromise feed efficiency, however, that hypothesis is not supported by the phenotypes characterized in this study. Nonetheless, there remains a possibility that CD4^+^ T cells in HE cows possess an enhanced capability and metabolic fitness to utilize nutrients for the synthesis of biomolecules required for synthesis of cytokines and cell proliferation compared to LE cows.

It is noteworthy to mention that immune responsiveness varies according to stage of lactation in dairy cows^31^. Eder et al.^32^ conducted a study to determine the relationships between CD4^+^ T lymphocytes and stage of lactation in healthy Holstein cows. The authors showed that mitochondrial function did not differ in stimulated and non-stimulated CD4^+^ T lymphocytes across different stages of lactation, which is contradictory to our results; however, rates of glycolytic capacity and glycolytic reserve in activated CD4^+^ T lymphocytes were greater than non-activated lymphocytes throughout the lactating and dry periods in dairy cows^32^. Yet, Shuster et al. (1996) demonstrated that cows in mid-lactation displayed better immune responsiveness against *E. coli* infections compared to those in early lactation. It is known that in early lactation, dairy cows experience considerable lipid mobilization, resulting in increased blood concentration of saturated fatty acids, and those fatty acids are also incorporated into cellular membranes of PBMC^33^. The increased mobilization of saturated fatty acids and phospholipids might change fatty acid composition of immune cells, which might play a role in the immunosuppressive profile in early lactation cows^33–35^. Conversely, mid-lactating cows are considered immunocompetent as they achieve a balance between milk production and energy intake. The findings from this study suggest that T lymphocytes in mid-lactating HE cows are persistent and probably metabolically more capable of carrying out a durable immune response, which might involve the synthesis of cytokines, rapid cell proliferation, and generating memory-like T cells. While these responses were not elucidated in this study, future research should be targeted to characterize the disease and immune functions according to divergent RFI in dairy cows.

Although the research presented herein supports that there were differences in energetics in immune cells isolated from LE or HE cows, it is important to note that this study was not designed to quantify the energetic cost or savings of these differences. One aspect that is worthy of mentioning is that energy requirements in pregnant or non-pregnant cows, especially in the late lactation, diverge substantially. Although the goal of the present study was not to measure the link between feed efficiency and reproduction, we observed no associations between feed efficiency and pregnancy rate, services per conception, or days open postpartum. Data on reproduction was only collected to assure that all cows in this study were not only around similar stage of lactation but also had similar stage or requirements of pregnancy which should not be confounded with energy requirements of immune system. Further investigation is necessary to determine if these immune cell energetic differences contribute to individual cow variance in RFI as an appreciable energy sink that should be accounted for, or if the difference only contributes to potential differences in immune readiness or responsiveness to immune challenges. Given the fact that cows in this study did not have immune challenges, and the differences observed both without and with activation of CD4^+^ T lymphocytes *in-vitro*, these data support that immune cell nutrient use may be more efficient in HE cows.

## Conclusions

This study demonstrated that HE cows had an increased expression of CD62L^+^ cells within CD8^+^ T lymphocytes and CD21^+^ B lymphocytes compared to LE cows. The increased expression of CD14^+^ cells were observed within CD11b^+^ monocytes in HE cows compared to LE cows. Even though cows in this study did not have immune challenges, an increased expression of T-cells, B-cells, and monocytes suggest a state of immune readiness, which may serve a crucial role in mounting an effective immune response against pathogens. Additionally, the increased metabolic activity and SRCR observed in CD4^+^ T lymphocytes in HE cows specifically demonstrates an enhanced metabolic fitness and immune cells’ capacity to respond to stimuli, such as mitochondrial stress. The results derived from this study imply that T lymphocytes in mid-lactation HE cows exhibit sustained activity and are likely to possess enhanced metabolic capabilities to sustain a robust and enduring immune response. This enhanced response may potentially include synthesis of cytokines or chemokines, rapid cell proliferation, and the formation of T-cells akin to memory cells. While these specific immune responses and whole animal energetic cost of immune readiness as a potential energy sink were not elucidated in this study, our findings suggest the need for future research endeavors to delve into characterizing disease patterns and immune functions based on divergent RFI when subjected to an immune challenge in dairy cows.

## Materials and methods

All animal use and handling protocols were approved by the University of Wisconsin-Madison College of Agricultural and Life Sciences Animal Care and Use Committee, protocol A006420-A03. All experiments were performed in accordance with relevant guidelines and regulations and authors complied with the ARRIVE guidelines.

### Study design and sample size calculations

The design of the present study had 2 components. The first component was a prospective cohort study aimed to characterize immune cells phenotype and neutrophil function in LE and HE dairy cows. The sample size was determined based on proportions of lymphocytes and monocytes to detect a 2.5% difference with a standard deviation of 2% in their proportions between LE and HE cows. The sample size was calculated using JMP Pro (Version 15. SAS Institute Inc., Cary, NC) with a two-tailed test (α = 0.05; β = 0.20). Under these assumptions, the sample size calculated was a minimum of total 23 cows. To account for potential attrition during the study, a sample size of at least 28 cows (LE; n = 14, and HE; n = 14) was used in the first component of the study.

The second component was a randomized complete block design to evaluate the metabolic function of CD4^+^ T lymphocytes under resting and activated states in LE and HE dairy cows. The sample size was calculated based on basal respiration and maximal respiration as reported previously^14,32^. Based on an anticipated difference of 30 pmol/min in the maximal respiration between groups with a standard deviation of 20 pmol/min, a sample size of total 18 cows were determined to be sufficient for achieving 80% power at an α level of 0.05. To account for potential attrition, a sample size of at least 24 cows (LE; n = 12, and HE; n = 12) was used in the second component of this study. The LE and HE cows used in the second component of this study belonged to the same cohort of cows that were enrolled in the first component of the study. The rationale to enroll 24 cows in the second component instead of all 28 cows was because 2 LE cows were sold, 1 HE cow experienced abortion, and 1 HE cow had lameness.

### Cows, housing, and feeding management

In the first component of the study, sixty-four multiparous Holstein dairy cows were enrolled at mean (± standard deviation) 93 ± 22 days in milk (DIM) in free stalls at the Emmons Blaine Dairy Cattle Center (Arlington, WI, USA) for 7 weeks. Cows were allowed ad libitum access to feed and water during the study. Feed intake was recorded daily for each cow with electronic roughage intake control feeding bins (Hokofarm Group, Marknesse, The Netherlands) for the first 7 weeks. Feed was offered twice daily at 0900 and 1500 h. The diet was fed as a total mixed ration (TMR), and the chemical composition of the diet is presented in Table 1. Samples of individual feedstuffs and the TMR were sampled weekly and used for DM and nutrient composition analyses as previously described^3^. Briefly, weekly samples were dried at 105 °C in a forced-air oven for 24 h to determine weekly DM of the TMR. Weekly feedstuff samples were dried at 55 °C in a forced-air oven for 48 h, ground through a 1 mm screen (Wiley Mill, Arthur H. Thomas, Philadelphia, PA, USA), and composited across the entire study for nutrient composition analysis. Individual composited samples were analyzed at a commercial laboratory (Dairyland Laboratories, Inc., Arcadia, WI, USA). Details on feedstuff analysis are provided elsewhere^36^.

### Body weight and body condition score

Cows were weighed in the morning using a walk-through calibrated electronic scale on 3 consecutive days, during week 1, 4, and 7 of the study. Obtained body weights were averaged into single means for the beginning, middle, and end of the study and the change in body weight was calculated as described previously^3^. Concurrent with body weight, body condition was scored by two individuals using a 1 to 5 scale with increments of 0.25 units^37^, as depicted in the Elanco body condition score chart^38^ and the mean value was calculated for the beginning, middle, and end of the study.

### Measurement of yields of milk and milk components

Mid-lactating cows (first component of the study) were milked twice daily at 0400 and 1500 h. Late lactating cows (second component of the study) were milked at 0800 and 1900 h. Throughout both components of the study, yields of milk were recorded electronically at each milking. Milk samples were taken weekly at four consecutive milkings and analyzed for milk composition at a commercial laboratory (AgSource, Menominee, WI, USA) as previously described by Oliveira et al.^39^. Yields of milk component for specific milking sessions were calculated by multiplying the milk yield by the milk composition for that milking session, and daily yields of the components were calculated by summing successive afternoon and morning milkings. The NE_L_ required for milk synthesis was calculated according to yields of fat, protein, and lactose, based on NASEM^40^ as follows: NE_L_ secretion in milk (Mcal/d) = (9.29 × fat yield) + (5.63 × true protein yield) + (3.95 × lactose yield), with yields of milk components in kilograms per day. Energy-corrected milk yield (ECM; kg/d) was calculated as [(0.327 × milk yield (kg)) + (12.95 × milk fat (kg)) + (7.65 × milk protein (kg))] according to Orth^41^. The content of NE_L_ of diet (Mcal/kg of DM) fed was calculated using the analyzed nutrient content of ingredients and adjusted by the mean DMI observed in this study. The NE_L_ intake (Mcal/d) was calculated as NE_L_ content of the diet ⨉ DMI (kg/d).

### Prospective cohorts formation according to residual feed intake calculation

Residual feed intake was calculated based on the performance data collected during the first 7 weeks of the study to align with body weight and body condition score measurements. It has been shown that a minimum 45-day measurement window can effectively approximate the RFI for the entire lactation phase^42,43^. Residual feed intake was calculated by regression using R (version 4.2.2, R Core Team 2022) with the following model:

$${\text{DMI}}_{{\text{i}}} = \, \mu \, + \, \beta_{{1}} \times {\text{ MilkE}}_{{\text{i}}} + \, \beta_{{2}} \times {\text{ MBW}}_{{\text{i}}} + \, \beta_{{3}} \times \, \Delta {\text{BW}}_{{\text{i}}} + \, \beta_{{4}} \times {\text{ DIM}}_{{\text{i}}} + {\text{ RFI}}_{{\text{i}}} ,$$

where DMI_i_ = the observed average DM intake (kg/day) of the ith cow; µ = overall mean; MilkE_i_ = secreted milk energy of the ith cow; MBW_i_ = metabolic body weight (BW^0.75^) of the ith cow; ΔBW_i_ = daily change in body weight of the ith cow; and DIM_i_ = midpoint DIM of the ith cow^44^. Regression coefficients β_1_, β_2_, β_3_, and β_4_ corresponded to secreted milk energy, BW^0.75^, change in body weight, and midpoint days in milk, respectively. The random residual, RFI_i_, represents the RFI phenotype of the ith cow. Of the 64 available cows, 5 were not considered eligible for RFI analysis due to severe health issues that had visible effects on feed intake and/or milk yield. Thereafter, based on the RFI values, 59 cows were ranked from LE to HE, and the top and bottom 24% of cows were selected as a cohort of 14 LE and 14 HE to characterize the immune cells phenotype and neutrophils function in week 8 of the study.

### First component of study: blood sampling and immune cells phenotyping

Blood was sampled from the coccygeal vein of 28 cows (LE; n = 14, and HE; n = 14) at mean (± standard deviation) 149 ± 25 DIM immediately after the morning milking from LE and HE cows. A total of 10 mL of blood was collected from each cow in a vacutainer tube containing K_2_EDTA to prevent clotting (Vacutainer, Becton Dickson, Franklin Lakes, NJ) and transferred to the lab in ice within 35 min after collection. Blood tubes were centrifuged at 1200 × *g* for 15 min at room temperature and the buffy coat was harvested and transferred into a 50 mL sterile tube. The buffy coat was diluted with 15 mL of phosphate buffered saline (PBS). The PBMC were isolated by density gradient separation using Histopaque-1077 (10,771, Sigma-Aldrich, St. Louis, MO) and washed twice in PBS. Red blood cells were removed by hypotonic lysis using double-distilled water. Purified PBMC were resuspended in PBS containing 0.5% bovine serum albumin (BSA; A3294, Sigma-Aldrich, St. Louis, MO).

Briefly, PBMC were transferred to two identical microcentrifuge tubes, centrifuged at 600 ×* g* for 5 min and the supernatant was discarded. One aliquot was used for T cells panels and incubated for 30 min at room temperature in the dark with 1 µg of anti-γδ (BOV2058, cell line: GB21A, isotype IgG2b, College of Veterinary Center, WSU, WA). Next, PBMC were washed twice with PBS supplemented with 0.5% of BSA (PBS-BSA) and incubated for an additional 30 min at room temperature in the dark with 0.05 µg of anti-CD4 (MCA1653A700, clone CC8, Bio-Rad Laboratories, Inc., Hercules, CA), 3 µg of anti-CD8 (MCA837PE, clone CC63, Bio-Rad Laboratories, Inc., Hercules, CA), 0.1 µg of anti-CD21 (MCA1424F, clone CC21, Bio-Rad Laboratories, Inc., Hercules, CA), 0.5 µg of APC (1090-11L, Isotype Goat IgG, SouthernBiotech, Birmingham, AL), 1 µg of anti-CD62L (WS0515B-100, clone BAQ92A, Kingfisher Biotech, Inc., Saint Paul, MN) conjugated with Pacific Blue™ Antibody Labeling Kit (P30013, Thermo Fisher Scientific Inc., Waltham, MA), and 1 µL of LIVE/DEAD™ Fixable Aqua Dead Cell Stain Kit (L34965, Thermo Fisher Scientific Inc., Waltham, MA) in 560 µL of PBS-BSA. After that, PBMC were washed twice with PBS containing 0.5% BSA (PBS-BSA), filtered in a cap w/ 35 µm strainer mesh, and transferred to a flow cytometry tube.

The second aliquot was used for B cells and monocytes panels and incubated for 30 min at room temperature in the dark with 0.05 µg of anti-CD11b (MCA1425A647, clone CC126, Bio-Rad Laboratories, Inc., Hercules, CA), 1µL of anti-CD14 (MCA2678PE, clone CC-G33, Bio-Rad Laboratories, Inc., Hercules, CA), 0.1 µg of anti-CD21, 1 µg of anti-CD62L and 1 µL of live/dead™ Fixable Aqua Dead Cell Stain Kit in 450 µL of PBS-BSA. After that, PBMC were washed twice with PBS-BSA, filtered in a cap with 35 µm strainer mesh, and transferred to a flow cytometry tube.

The stained cells were re-suspended in 1 mL of PBS before running on an Attune NxT Flow Cytometer (Thermo Fisher Scientific, Waltham, MA). Flow cytometry data was analyzed with FlowJo (FlowJo LLC, Ashland, OR), using fluorescent minus one sample to establish appropriate gating. Each antibody and conjugating fluorochrome was titrated to determine the optimal concentration for staining, and compensation beads (UltraComp eBeads Plus microspheres, ThermoFisher) were used to calculate spillover values and set appropriate voltages and gating parameters. The viability of cells was ≥ 94%, and data were analyzed on live and single cell events using FlowJo software (Version 10.0.7, Treestar, Palo Alto, CA).

### First component of study: blood sampling and neutrophils function

A total of 10 mL of blood was sampled from the coccygeal vein of 28 cows (LE; n = 14, and HE; n = 14) cows at 149 ± 25 DIM immediately after the morning milking. Blood was collected in vacutainer tube containing sodium heparin to prevent clotting (Vacutainer, Becton Dickson, Franklin Lakes, NJ) and transferred to the lab at room temperature within 35 min after collection. Eight mL of blood was collected and transferred into a 50 mL sterile tube with 20 mL of PBS. Neutrophils were isolated by density gradient separation using 8 mL of Histopaque-1077 (10771, Sigma-Aldrich, St. Louis, MO). Plasma, PBMC, and histopaque were removed, and red blood cells were removed by hypotonic lysis using double-distilled water. Purified neutrophils were resuspended in PBS containing 10% of fetal bovine serum (FBS; F2442, Sigma-Aldrich, St. Louis, MO), counted with 0.08% solution of trypan blue (T8154, Sigma-Aldrich, St. Louis, MO) using a counting chamber, and diluted to a concentration of 5.0 × 10^6^ cells/mL.

In order to perform the phagocytosis assay in neutrophils, zymosan-activated serum (ZAS) was prepared after incubating 100 mg of zymosan (Z2849, Thermo Fisher Scientific Inc., Waltham, MA) with 10 mL of 10% FBS (Sigma-Aldrich, St. Louis, MO) for 60 min at 37 °C in a rotating rack. Thereafter, ZAS was centrifuged at 1600 rpm for 10 min, and stored at -80 °C after filtration. Neutrophils at a density of 1 × 10^6^ were pipetted in two tubes. One tube was incubated with 1 × 10^7^ beads, i.e. TransFlouSphers Carboxylate-Modified Microspheres (T8880, Thermo Fisher Scientific Inc., Waltham, MA), along with 50 µL of ZAS, and 200 µL of PBS/FBS, and represented the positive control. The second tube was only incubated with 50 µL of ZAS supernatant in 200 µL of PBS/FBS (200 µL of PBS containing 10% of FBS) and served as a negative control. Both tubes were incubated for 30 min at 37 °C in a shaking water bath protected from the light. After incubation, neutrophils were washed twice with PBS and resuspended in 400 µL of PBS/FBS.

In order to perform the oxidative burst assay, 1 × 10^6^ neutrophils were pipetted in two tubes. One tube was incubated with 200 µL of phorbol 12-myristate 13-acetate (PMA i.e. 2 µg/mL (J63916.MA, Thermo Fisher Scientific Inc., Waltham, MA), 0.02 mM of 2′,7′-dichlorofluorescin diacetate (H_2_DCFDA; D6883, Sigma-Aldrich, St. Louis, MO), and 200 µL of PBS/FBS and represented the positive control. The second tube was incubated with 0.02 m*M* of H_2_DCFDA, and 400 µL of PBS/FBS, and served as a negative control. Both tubes were incubated for 30 min at 37 °C in a shaking water bath protected from the light. Median fluorescence intensity was measured using flow cytometer to assess the phagocytosis and oxidative burst capacities in neutrophils.

### Second component of study: bioenergetic measures in CD4^+^ T lymphocytes

In the second component of this study, a total of 24 cows (LE; n = 12, and HE; n = 12) from an already established cohort of LE and HE cows, were enrolled in the first component of this study. Data on health events were stored and extracted via archived computer backup files from an on-farm dairy management software program (DairyComp 305; Valley Agricultural Software). All 24 cows were healthy and did not experience any health problems such as mastitis, pneumonia, lameness, and indigestion at least 40 days before conducting the second component of this study. All 24 cows were blocked according to their DIM. Twelve cows from the already established cohort of LE and twelve cows from HE cohort were enrolled again at mean (± standard deviation) 234 ± 22 DIM. The rationale of this component of the study was to evaluate the metabolic function of CD4^+^ T lymphocytes under resting and metabolically activated state in LE and HE dairy cows.

### Second component of study: isolation and purification of CD4^+^ T cells

During the second component of the study, cows were grouped with late-lactation cows and blood samples were collected prior to milking due to the later milking time of late-lactation cows. A total of 60 mL of blood was sampled from the coccygeal vein of 24 cows at mean (± standard deviation) 234 ± 22 DIM before the morning milking at approximately 0630 h. Blood was collected in vacutainer tubes containing K_2_EDTA to prevent clotting (Vacutainer, Becton Dickson, Franklin Lakes, NJ) and transferred to the lab at room temperature within 35 min after collection. Blood tubes were centrifuged at 1200 ×* g* for 15 min at room temperature and buffy coat was harvested and transferred into a 50 mL sterile tube. The buffy coat was diluted with 15 mL of PBS. Peripheral blood mononuclear cells were isolated by density gradient separation using Histopaque-1077 (Sigma-Aldrich, St. Louis, MO) and washed twice in PBS. Red blood cells were removed by hypotonic lysis using double-distilled water. Purified PBMC were resuspended in PBS-BSA. Peripheral blood mononuclear cells were counted using an automatic cell counter (Countess II FL, Invitrogen, Waltham, MA), and the viability of cells was assessed by diluting cell suspension with 0.08% (vol/vol) solution of trypan-blue (T8154, Sigma-Aldrich) in a 1:1 dilution.

The CD4^+^ T lymphocytes were isolated using magnetic cell separation (Miltenyi Biotec, Bergisch Gladbach, Germany) according to manufacturer instructions. Briefly, aliquots of 1 × 10^7^ of PBMC were first incubated for 30 min at room temperature in the dark with 5 µL of anti-CD4 (WS0562B-100, clone ILA11A, Kingfisher Biotech, Inc., Saint Paul, MN) in 100 µL PBS-BSA. Thereafter, PBMC were washed twice with PBS-BSA and incubated for an additional 15 min at 4 °C by adding 20 µL of MACs IgG2a + b magnetic beads (130–047-202, Miltenyi Biotec) and 80 µL of PBS-BSA. Peripheral blood mononuclear cells were washed again with PBS-BSA, and the bead-CD4^+^ T lymphocytes complex was extracted with a LS magnetic-activated cell sorting column (130-042-401, Miltenyi Biotec) according to the manufacturer’s instructions. During the first incubation of PBMC with the ILA11A antibody, PBMC were also incubated with 10 µL of anti-CD4 conjugated to Alexa Fluor 700 (MCA1038A700, Biorad, Berkley, CA) in order to determine the purity of the isolated CD4^+^ T lymphocytes population. The purity of CD4^+^ T lymphocytes was assessed by flow cytometry (Attune NxT Flow Cytometer, Thermo Fisher Scientific, Waltham, MA). The viability of sorted CD4^+^ T lymphocytes was assessed using 0.08% trypan blue solution in an automatic cell counter (Countess II FL, Invitrogen). The CD4^+^ T lymphocytes were counted to prepare two aliquots containing 2.4 × 10^6^ cells in each aliquot for downstream Seahorse analysis.

### Second component of study: CD4^+^ T lymphocytes persistence assay using XF HS mini SeaHorse

The Seahorse analysis was performed to evaluate bioenergetics measures in CD4^+^ T lymphocytes using a XFp T Cell Metabolic Profiling Kit (103771-100, Agilent technologies, Inc., Santa Clara, CA) in a Seahorse XF HS Mini Analyzer. The day prior to analysis, the Agilent Seahorse XFp Extracellular Flux Cartridge (103022-100, Agilent Technologies, Inc.) was hydrated according to manufacturer’s instructions and placed in a non-CO_2_ incubator overnight at 37 °C. Additionally, approximately 5 mL of XF Calibrant media (103022–100, Agilent Technologies, Inc.) for the hydration of each utility plate of cartridge and XFp PDL Miniplate (103722-100, Agilent Technologies, Inc.) were placed in a non-CO_2_ incubator overnight at 37 °C. On the day of analysis, the standard Seahorse XF RPMI assay medium was prepared by supplementing 19.4 mL of Seahorse XF RPMI medium (103576-100, Agilent technologies, Inc.) with glucose (103577-100, XF 1.0 M Glucose Solution, Agilent technologies, Inc.), pyruvate (103578-100, XF 100 mM Pyruvate Solution, Agilent technologies, Inc.), and glutamine (103579-100, XF 200 mM Glutamine Solution, Agilent technologies, Inc.) solutions to achieve a final concentrations of 10 mM, 1mM, and 2mM, respectively, and stored at 37 °C throughout the assay as reported previously^14^.

The isolated CD4^+^ T lymphocytes from each LE and HE cow were split into two tubes and randomly assigned to incubate with either 250 µL of XF RPMI assay medium to maintain cells in the resting state or control (CON) or treated with a combination of 140 µL of Seahorse XF RPMI assay medium, 100 µL of PMA (J63916-M, Thermo Fisher Scientific) at 20 ng/mL, and 10 µL of ionomycin (IMY; I24222, Invitrogen) at 1﻿µg/mL (PMA + IMY) for 2 h at 37 °C. The rationale for incubating CD4 + T lymphocytes with a combination of PMA + IMY (metabolically active state) was to induce immune activation and promote high metabolic activity in immune cells. This methodology stemmed from the observed increase of cytokine production by immune cells when exposed to this combination^45^. Each XFp PDL miniplate represented 1 LE and 1 HE cow containing non-stimulated and stimulated CD4^+^ T lymphocytes, separately. After 2 h incubation of CD4^+^ T lymphocytes with treatments, 50 µL of CD4^+^ T lymphocytes suspension from each tube was added in triplicate to have 4.0 × 10^5^ live cells density per well in XFp PDL miniplate^32^. Thereafter, 50 µL of Seahorse XF RPMI assay medium was added to each well, and the moats around each well were filled with 200 µL of warm sterile water. The XFp PDL miniplate was centrifuged at 200 × *g* for 1 min to allow cells to attach to the bottom of the wells, and additional 100 µL of warm XF RPMI assay medium was added in each well. Thereafter, the XFp PDL miniplate was incubated at 37 °C in a non-CO_2_ incubator for 60 min prior to performing the T cells persistence assay. On the day of the analysis, the XFp Extracellular Flux Cartridge was removed from the incubator and rehydrated with pre-warmed XF Calibrant media according to manufacturer's instructions. The cartridge was then placed back into the non-CO_2_ incubator at 37 °C for at least 60 min prior to adding the port injection substrates. The port injection substrates in XFp T cell metabolic profiling kit included complex V inhibitor (oligomycin), a protonophore uncoupler (BAM 15), and complex I and complex III inhibitors (rotenone and antimycin A, respectively). Each reagent was resuspended with 0.5 mL of warm XF RPMI assay medium to achieve final concentrations of 13.5 µM oligomycin, 25 µM BAM 15, and 5.5 µM rotenone and antimycin A. 25 µL of oligomycin, BAM 15, and mix of rotenone and antimycin A were added in ports A, B, and C, respectively, for each well of XFp PDL miniplate. The rationale to add these reagents in injection ports was to induce mitochondrial stress in CD4^+^ T lymphocytes and measure the bioenergetic changes in those cells. After injection substrates were added to each port, the cartridge was placed in the XF HS Mini Seahorse for calibration. Once the cartridge was calibrated, the XFp PDL miniplate was inserted into the XF HS Mini Seahorse machine for analysis to perform T cells persistence assay. The assay was conducted in triplicate, and two wells within each PDL miniplate were designated for quality control (lacking CD4 + T lymphocytes) purposes.

Data were exported and quality of data examined using the raw excel outputs and Seahorse Wave Pro Software (version 10.1.0, Agilent Technologies, Inc. 2021). During this assay, mitochondrial function kinetics were recorded in real time, measuring OCR (pmol/min) as a marker for oxidative phosphorylation, whereas cytoplasmic pH changes were monitored in real time measuring ECAR (mpH/min) as an indicator of cellular glycolysis. Additionally, PER (pmolH^+^/min) was measured which represents a quantitative assessment of extracellular acidification. The measures of bioenergetics were calculated using Seahorse Analytics (version 1.0.0-570, Agilent Technologies, Inc., 2023) and included basal mitochondrial ATP production rate (pmol/min), basal glycolytic ATP production rate (pmol/min), basal total ATP production rate (pmol/min), basal mitochondrial respiration rate (pmol/min), maximal mitochondrial respiration rate (pmol/min), and SRCR (pmol/min). The bioenergetic measures for each plate were averaged across each triplicate.

### Second component of study: reproductive management and performance

Data on reproductive management and performance were entered, stored and extracted via archived computer backup files from an on-farm dairy management software program (DairyComp 305; Valley Agricultural Software). All 24 cows were synchronized using standardized Double-Ovsynch protocol starting 78 ± 3 day postpartum, and AI were performed in the morning. Cows which returned to estrus after an AI were again inseminated on the same day. Cows that were re-inseminated before pregnancy diagnosis were considered not pregnant to the previous AI. Pregnancy was diagnosed by transrectal ultrasonography on day 32 ± 3 after each AI based on the presence of an amniotic vesicle with an embryo with heartbeat. Pregnant cows on day 32 after AI were re-evaluated for pregnancy on days 70 ± 3 after AI. Data on total number of AI performed for a particular cow to achieve pregnancy were also collected. Calculation of days open included AI that occurred up to 300 days postpartum, and pregnancy was based on the diagnosis on day 74 after AI.

### Statistical analysis

Normality of residuals and homogeneity of variance were examined for each continuous dependent variable analyzed after fitting the statistical model. Responses such as OCR, ECAR, and PER violated the assumptions of normality and were subjected to power transformation according to the Box-Cox procedure^46^ using PROC TRANSREG in SAS (SAS/STAT, SAS Institute Inc., Cary, NC); however, the interpretation of data did not change after transformation. Therefore, the least squares of the means (LSM), and respective standard errors of the means (SEM) were presented on original scale to avoid back transformation of SEM. The first component of the study focusing on immune cells characterization in LE and HE cows followed a prospective cohort design; however, the data on energy characteristics and productive performance was analyzed retrospectively to determine the RFI status of cows. Data were analyzed by mixed-effects models using the MIXED procedure of SAS (ver. 9.4, SAS/STAT, SAS Institute Inc., Cary, NC). The statistical models included the fixed effects of feed efficiency (LE vs. HE), time of measurement (week), the interaction of feed efficiency and time, and the random effect of residuals.

The second component of the study followed a randomized complete block design. Cows were ranked by DIM, from the smallest to the largest value, and then one cow from each group (1 LE and 1 HE) were assigned a block. Within each block, the CD4^+^ T lymphocytes were isolated from each cow and split into two tubes, which were assigned randomly to CON (resting state) or PMA + IMY (metabolically activated state) treatments. Each XFp PDL miniplate represented 1 LE and 1 HE cow containing non-stimulated and stimulated CD4^+^ T lymphocytes, separately, thus, cow was considered the experimental unit. Data comprised of resting and stimulated states of CD4^+^ T lymphocytes were analyzed separately. Data were analyzed by mixed-effects models using the MIXED procedure of SAS (ver. 9.4, SAS/STAT, SAS Institute Inc., Cary, NC). The statistical models included the fixed effects of feed efficiency (LE vs. HE), time (minutes) of measurement, and the interaction between feed efficiency and time, and the random effects of block and cow nested within feed efficiency status. In all statistical models with repeated measures, the REPEATED statement was used for dependent variables measured over time. The cow nested within feed efficiency cohort was the error term for testing the association of feed efficiency. The response variables had equal spacing between measurements; therefore, autoregressive 1 was the most selected covariance structure. When an interaction between feed efficiency and time resulted in *P* < 0.10, then means of the two feed efficiency cohorts at different time points were partitioned using the SLICE command of SAS (SAS Institute Inc.). The Kenward-Roger method was used to approximate the denominator degrees of freedom to compute the *F* tests.

Binary data such as pregnancy per AI were analyzed by logistic regression using the GLIMMIX procedure of SAS (SAS/STAT, SAS Institute Inc.) fitting a binary distribution. The statistical model for a binary response with a single measurement per cow was exactly the same as those described for continuous variables previously.

Pearson correlation coefficient analysis was used to test the relationship between RFI and bioenergetic measures in non-stimulated and stimulated CD4^+^ T lymphocytes. The resulting correlation matrix was visualized in a heatmap format generated by the corrplot package of R [Corrplot: visualization of a correlation matrix; R package version 4.1.2. 2021]. Correlation coefficients were classified as strong (r > 0.75), moderate (0.50 < r ≤ 0.75), and weak (0.25 < r ≤ 0.50). Evidence of statistical significance against the null hypothesis was considered at *P* ≤ 0.05, and tendency was considered at 0.05 < *P* ≤ 0.10.

## Data Availability

The data that support the findings of this study are available, upon reasonable request, from the corresponding author (Heather M. White).
